# Microgravity and Space Medicine

**DOI:** 10.3390/ijms22136697

**Published:** 2021-06-22

**Authors:** Daniela Grimm

**Affiliations:** 1 Department of Biomedicine, Aarhus University, Ole Worms Allé 4, 8000 Aarhus C, Denmark; dgg@biomed.au.dk; Tel.: +45-8716-7693; Fax: +45-8612-8804; 2 Department of Microgravity and Translational Regenerative Medicine, Clinic for Plastic, Aesthetic and Hand Surgery, Otto von Guericke University, Universitätsplatz 2, 39106 Magdeburg, Germany; 3 Research Group “Magdeburger Arbeitsgemeinschaft für Forschung unter Raumfahrt- und Schwerelosigkeitsbedingungen” (MARS), Otto von Guericke University, 39106 Magdeburg, Germany

This Special Issue (SI), “Microgravity and Space Medicine”, covers research articles and reviews focusing on gravitational biology, cancer research and space medicine. It includes publications investigating the effects of altered gravity conditions on mammalian cells and humans during real microgravity (r-μg) on the International Space Station (ISS) and parabolic flights (PFs). Furthermore, it addresses the impact of simulated microgravity (s-μg) on human cells, cancer cells and animals.

In the near future, humans will return to the Moon and start expeditions to Mars and other planets. In addition, there will be an increase in space tourism, which will lead to a high number of manned spaceflights. A long-term stay in space can influence the health of space travelers and result in various health problems, such as dysregulation of the immune system, bone loss, muscle atrophy, cardiac problems or impaired wound healing [1,2,3], among others.

Space provides an extreme environment of stressors (e.g., microgravity and cosmic radiation) that humans, mammals and cells do not experience on Earth. Cells in space on board the ISS, unmanned spacecraft, satellites and other carriers exhibited a large number of changes and develop a range of molecular biological responses [4,5,6,7]. Short-term stays in real (r-) μg were possible through PF missions. PFs are dedicated to scientific experiments with cells, small organisms or humans, or material science and engineering studies (e.g., function of hardware containers, microscopes, etc.) [8].

Several devices were designed to simulate μg on Earth. The European Space Agency (ESA) has acknowledged several μg-simulators as so-called ESA ground-based facilities (GBF). Examples are the random positioning machine (RPM), the 2D and 3D clinostat for μg-simulation or centrifuges for hyper-g experiments like the Short-Arm Human Centrifuge at the German Aerospace Center (Deutsches Zentrum für Luft- und Raumfahrt, DLR) in Cologne, Germany and the Large Diameter Centrifuge at ESA/ESTEC (Noordwijk–The Netherlands). The NASA Microgravity Simulation Support Facility at Kennedy Space Center offers slow rotating clinostats, rotating wall vessel (RWV) bioreactors and, among others, high aspect ratio vessels and slow turning lateral vessels. Furthermore, 3D clinostats and RPM devices are available for researchers.

An animal model suitable for the simulation of μg is the worldwide-used hindlimb unloading model (HU) [9]. The HU model is used to study the impact of the spaceflight environment on integrated physiologic, organ-specific and mechanistic responses.

The Stress Immunity Pathogens laboratory at the University of Lorraine provides the Gravitational Experimental Platform for Animal Models (GEPAM), which comprises three aquatic rotors, an RPM and a rodent large-radius rotor. Bonnefoy et al. have introduced and published the GEPAM facility in this SI [10].

This SI published 11 research articles (Table 1) investigating the impact of r-μg on cells [11,12] and humans [13], as well as of s-μg on animals [14,15,16] and cells [17,18,19,20,21].

In addition, the SI covers five reviews (Table 2) [10,22,23,24,25].

These 16 excellent papers were published as detailed in Table 1 and Table 2.

This SI covered two studies investigating human cells on the ISS in space [11,12]. Adult and neonatal cardiovascular progenitors were cultured for 30 d on the ISS. The authors showed an induction of signaling pathways, supporting proliferation and survival by spaceflight together with transcripts related to cell cycle re-entry, cardiovascular development and oxidative stress [11]. The CellBox-1 experiment focused on human thyroid cancer cells flown to the ISS during the SpaceX CRS-3 cargo mission [26]. The initial results show differences in the number of secreted exosomes released from the thyroid cancer cells and in the distribution of subpopulations regarding their surface protein expression [12].

One study investigated the short-term effects of PF maneuvers on the human organism (enrolled 14 healthy participants) [13]. The authors measured different heart failure biomarkers and found a reduction in cardiac stress and an influence of μg on the immune system through impairment and a decrease in inflammatory activation [13].

Another research team used the HU model and focused on muscle atrophy (MA) during mechanical unloading. MA in space can be supported by insufficient stress response and elevated oxidative stress. They studied F344 rats and showed that Nox2 inhibition regulates the stress response, and unloading-induced soleus fiber atrophy was significantly attenuated by gp91ds-tat [14].

Ogneva et al. studied *Drosophila melanogaster* sperm under s-μg (RPM) conditions and a hypomagnetic field. They focused on sperm motility and cell respiration of the fruit fly [15] and demonstrated that the motility of the tail of the sperm of *Drosophila melanogaster* increases under RPM-exposure and decreases under hypomagnetic conditions [15]. The second paper by Ogneva et al. [16] reported on the sperm motility of mice under RPM- and hyper-g exposure. The results indicated that hyper-g led to more significant changes than RPM-exposure: after 1 h, a reduction in speed of sperm movement was found and after 3 h, the number of motile cells started to decrease. RPM-exposure did not alter the speed of movement, but the motile spermatozoa decreased after 6 h of exposure. In parallel, changes in the structure of the microtubule cytoskeleton were found [16]. Furthermore, changes in the cytoskeleton were detected in many different cell types [6,27].

Nowadays, it is widely accepted that the cytoskeleton plays a key role in sensing changes in gravity [27]. Monti et al. [17] investigated normal MCF-10A breast epithelial cells and MCF-7 breast cancer cells and detected apoptosis after a three-day RPM-exposure in MCF-7 cells growing in form of 3D structures, in which major modifications of cytoskeleton components were observed. In addition, survival strategies differed between the cell types [17]. A further study investigated normal breast epithelial cells (MCF-10A) exposed to a 3D-clinostat [20]. The authors focused on the linker of nucleoskeleton and cytoskeleton (LINC) complex and showed that the nuclear shape and differential gene expression are both responsive to s-μg in a LINC-dependent manner [20]. Cytoskeletal changes were also detected in 3D multicellular aggregates when human chondrocytes were exposed to an RPM for 28 d [18]. The authors demonstrated that long-term μg created by RPM-induced 3D growth in human chondrocytes. The tissue-engineered spheroids without any scaffolds showed no signs of apoptosis and the typical cartilage morphology [18].

Furthermore, spaceflight has enormous effects on bone homeostasis and health in astronauts [2]. Murine MC3T3-E1 osteoblast cells were exposed to an RPM in the absence or presence of an antioxidant (6-hydroxy-2,5,7,8-tetramethylchroman-2-carboxylic acid (Trolox)). RPM-exposure led to morphological and metabolic alterations characterized by increased levels of reactive oxygen species and a slowdown of the proliferative rate. The antioxidant prevented these changes [21]. A concise review focused on the available countermeasures addressing bone loss in space conditions [22]. The authors discussed the physiology related to bone metabolism due to long-term space-exposure and evaluated the effectiveness of both pharmacological and non-pharmacological countermeasures [22].

Another study used systems biological methods and performed an integrative analysis of regulatory module and found associations of μg with dysfunctions of various systems and tumorigenesis [19]. Analysis of the skeletal system revealed that most of the genes and miRNAs in the subnetwork were involved in bone loss. The analysis of the relationship between μg (RWV) and 20 cancer types showed that most cancers might be promoted by μg [19].

Moreover, a paper reviewed the current knowledge about endosomes and lysosomes in both r-μg and s-μg [24]. μg activates the endo-lysosomal system, and thus induces bone loss, muscle atrophy and stem cell differentiation. An improved knowledge about the lysosomal adaptation in space can be beneficial in the search for new biomarkers or therapeutic approaches to several disease pathologies on Earth and in space [24].

In addition, Sun et al. [23] reviewed the current knowledge about the dysfunction of macrophages exposed to μg and discussed the mechanisms of these abnormal responses.

Finally, the review by Nassef et al. [25] summarized the available information about the impact of μg on breast cancer cells. Breast cancer is the most invasive cancer in women. “Fighting breast cancer requires to think outside-the-box” [25]. μg research is an important tool for the development of 3D in vitro model systems suitable for pharmacological drug screening or even discovering novel breast cancer medications.

Taken together, the 16 excellent publications included in this SI demonstrate novel findings in the field of “Microgravity and Space Medicine”. I would like to thank all the authors who supported this SI. I am convinced that the application of space research using the platforms for r-μg and devices for s-μg in combination with new molecular biological technologies will be helpful in the health protection of future space travelers who conquer space during deep exploration and will also be applicable in translational regenerative medicine on Earth.

## Figures and Tables

**Table 1:** Research articles contributed to the Special Issue “Microgravity and Space Medicine”.

Author	Title	Topics	Reference
Camberos V. et al.	The impact of microgravity and spaceflight on the human islet-1+ cardiovascular progenitor cell transcriptome	ISS experiment (30 d): transcriptome of adult and neonatal cardiovascular progenitors. Gene expression profile representative of an early-stage, dedifferentiated, stem-like state, regardless of age. Signaling pathways that support cell proliferation and survival were induced by spaceflight, along with transcripts related to cell cycle re-entry, cardiovascular development and oxidative stress.	[11]
Lawler J. M. et al.	Nox2 inhibition regulates stress response and mitigates skeletal muscle fiber atrophy during simulated microgravity	F344 rats: control (CON), hindlimb unloaded (HU) and hindlimb unloaded +7.5 mg/kg/day gp91ds-tat (HUG) groups Causal role for Nox2 in unloading-induced muscle atrophy, linked to preservation of HSP70, Nrf2 and sarcolemmal nNOS.	[14]
Wise P. M. et al.	Changes in exosome release in thyroid cancer cells after prolonged exposure to real microgravity in space	CellBox-1 experiment: human thyroid cancer cells (FTC-133) flown to the ISS during the SpaceX CRS-3 cargo mission. Differences in the number of secreted exosomes and in the distribution of subpopulations in regards to their surface protein expression; alteration of their population regarding the tetraspanin surface expression.	[12]
Monti N et al.	Survival pathways are differently affected by microgravity in normal and cancerous breast cells	Normal (MCF-10A) and cancerous (MCF-7) breast cells exposed for 24 h or 72 h to an RPM. After 72 h apoptosis detectable in MCF-7 cells. Organoid-like structures, major modifications of the cytoskeleton. Survival strategies differ between cell types.	[17]
Wehland M. et al.	Tissue engineering of cartilage using a random positioning machine	Human articular chondrocytes exposed to an RPM for 24 h up to 28 d. s-μg-exposed chondrocytes revealed 3D spheroids without any scaffolds. The tissue engineered spheroids showed the typical cartilage morphology	[18]
Yuan M. et al.	Integrative analysis of regulatory module reveals associations of microgravity with dysfunctions of multi-body systems and tumorigenesis	Human peripheral blood lymphocytes exposed to s-μg (Rotating Wall Vessel). 230 dysregulated TF-miRNA (transcription factor and microRNA) feed-forward loops (FFLs). Relationship between μg and 20 cancer types; most cancers might be promoted by μg.	[19]
Neelam S. et al.	Changes in nuclear shape and gene expression in response to simulated microgravity are LINC complex-dependent	Effects of s-μg (3D clinostat) on the nucleoskeleton and cytoskeleton (LINC) complex of human breast epithelial cells (MCF-10A). Nuclear shape and differential gene expression are both responsive to s-μg in a LINC-dependent manner, duration-dependently.	[20]
Ogneva I. V. et al.	*Drosophila melanogaster* sperm under simulated microgravity and a hypomagnetic field: motility and cell respiration	Effects of s-μg and hypomagnetic conditions for 1, 3 and 6 h on the sperm motility of *Drosophila melanogaster*. Application of oral essential phospholipids (500 mg/kg in medium). s-μg: increased sperm tail movement speed after 6 h, change in cellular respiration, and a similar effect with the administration of essential phospholipids; a change in the phosphorylation of motor proteins.	[15]
Ogneva I. V. et al.	Sperm motility of mice under simulated microgravity and hypergravity	Effect of s-μg (RPM) and hyper-g (2× g, centrifuge) on mouse sperm motility and underlying mechanisms. Hyper-g: after 1 h: reduced speed of sperm movement; after 3 h: number of motile cells began to decrease. μg: no change in movement speed; reduced motile spermatozoa after 6 h. Changes in the microtubule cytoskeleton.	[16]
Morabito C. et al.	Antioxidant strategy to prevent simulated microgravity-induced effects on bone osteoblasts	Murine MC3T3-E1 osteoblast cells exposed to the RPM with/without an antioxidant (6-hydroxy-2,5,7,8-tetramethylchroman-2-carboxylic acid -Trolox). Morphological and metabolic alterations, increased levels of reactive oxygen species and a slowdown of the proliferative rate. Trolox inhibited the RPM-induced effects on the cytoskeleton, proliferation and metabolism.	[21]
Jirak P. et al.	Dynamic changes of heart failure biomarkers in response to parabolic flight	PF: influence on heart failure biomarkers H-FABP, sST2, IL-33, GDF-15, suPAR and Fetuin-A. PF Results: 1. Reduction in cardiac stress induced by exposure to gravitational changes. 2. Influence of gravitational changes on proliferative processes and calcium homeostasis.	[13]

**Table 2:** Reviews contributed to the Special Issue “Microgravity and Space Medicine”.

Author	Title	Topics	Reference
Genah S. et al.	The effect of space travel on bone metabolism: considerations on today’s major challenges and advances in pharmacology	Health risk for space travelers: μg-induced bone loss. Pharmacological and non-pharmacological countermeasures: physical exercise, diet supplements and antiresorptive or anabolic drugs.	[22]
Bonnefoy J. et al.	Gravitational experimental platform for animal models, a new platform at ESA’s terrestrial facilities to study the effects of micro- and hypergravity on aquatic and rodent animal models	New ESA ground-based facility: Gravitational Experimental Platform for Animal Models (GEPAM). To study the effects of altered gravity on aquatic animal models (amphibian embryos/tadpoles) and mice. Comprises rotors for hyper-g-exposure (three aquatic rotors and one rodent rotor) and models to simulate μg (cages for mouse HU and an RPM.	[10]
Sun Y et al.	The emerging role of macrophages in immune system dysfunction under real and simulated microgravity conditions	Gene expression: μg changes the expression of cytokines. Mitogen-activated protein kinase (MAPK) signaling pathway is involved in μg-induced immune malfunction. Macrophages are involved in μg- induced immune-system dysfunction.	[23]
Johnson I. R. D. et al.	Implications of altered endosome and lysosome biology in space environments	Lysosomes: Role in the regulation of autophagy, immunity and the adaptation of the organism to changes in their environment. μg activates the endo-lysosomal system. Impact on bone loss, muscle atrophy and stem cell differentiation.	[24]
Nassef M. Z. et al.	Breast cancer cells in microgravity: New aspects for cancer research	Breast cancer cells show various changes in μg: altered proliferation, survival, migration and a less-aggressive phenotype. 3D spheroids.	[25]

## Abbreviations

D	Day
DLR	Deutsches Zentrum für Luft- und Raumfahrt
ESA	European Space Agency
FTC	Follicular thyroid cancer
H	Hour
HU	Hindlimb unloading
ISS	International Space Station
MAPK	Mitogen-activated protein kinase
MA	Muscle atrophy
NASA	National Aeronautics and Space Administration
PF	Parabolic flight
r-μg	Real microgravity
RPM	Random positioning machine
RWV	Rotating wall vessel
SI	Special Issue
s-μg	Simulated microgravity
3D	Three-dimensional
